# Characteristics and Sonophotocatalytic Activity of Natural Sphalerite under Ultrasonic (1.7 MHz) and UVA LED (365 nm) Irradiation

**DOI:** 10.3390/ma15155412

**Published:** 2022-08-05

**Authors:** Svetlana Popova, Victoria Tazetdinova, Erzhena Pavlova, Galina Matafonova, Valeriy Batoev

**Affiliations:** 1 Laboratory of Engineering Ecology, Baikal Institute of Nature Management SB RAS, 670047 Ulan-Ude, Russia; 2 Chemistry Department, Buryat State University, 670000 Ulan-Ude, Russia

**Keywords:** natural sphalerite, sonophotocatalysis, 1.7 MHz high-frequency ultrasound, 365 nm UVA LED, degradation

## Abstract

Naturally occurring sono- and photoactive minerals, which are abundant on Earth, represent an attractive alternative to the synthesized sonophotocatalysts as cost-effective materials for water and wastewater treatment. This study focuses on characterizing and evaluating the sonophotocatalytic activity of natural sphalerite (NatS) from Dovatka deposit (Siberia) under high-frequency ultrasonic (US, 1.7 MHz) and ultraviolet light-emitting diodes (UVA LED, 365 nm) irradiation towards degradation of 4-chlorophenol as a model organic pollutant. Since raw natural sphalerite did not exhibit a measurable photocatalytic activity, it was calcined at 500, 900 and 1200 °C. The natural sphalerite after calcination at 900 °C (NatS*) was found to be the most effective for sonophotocatalytic degradation of 4-chlorophenol, attaining the highest efficiency (55%, 1 h exposure) in the following row: UV < US ≈ UV/US ≈ US/NatS* < UV/NatS* < UV/US/NatS*. Addition of 1 mM H_2_O_2_ increased the removal to 74% by UV/US/NatS*/H_2_O_2_ process. An additive effect between UV/NatS* and US/NatS* processes was observed in the sonophotocatalytic system as well as in the H_2_O_2_-assisted system. We assume that the sonophotocatalytic hybrid process, which is based on the simultaneous use of high-frequency ultrasound, UVA light, calcined natural sphalerite and H_2_O_2_, could provide a basis of an environmentally safe and cost-effective method of elimination of organic pollutants from aqueous media.

## 1. Introduction

At present, heterogeneous photocatalysis has been recognized as a powerful tool for water and wastewater treatment. It is based on the generation of reactive oxygen species (ROS), such as hydroxyl radicals (•OH), upon photoexcitation of solid semiconductor materials in aqueous solution. Over the last decade, sonophotocatalysis (SPC), which simultaneously utilizes ultrasonic (US) and ultraviolet/visible (UV/VIS) radiation, has emerged as a promising advanced oxidation process for degrading organic pollutants in water. SPC represents a considerable interest in view of enhanced ROS generation, intensification of oxidative reactions and, finally, occurrence of a synergistic effect in terms of accelerated degradation. It is known that •OH oxidize many organic pollutants with high second-order rate constants often in the range > 5 × 10^9^ M^−1^ s^−1^. As such, research on sonophotocatalytic degradation of organic pollutants has been intensified and comprehensively reviewed [1,2,3]. However, it should be emphasized that the synthesized photocatalysts, such as TiO_2_ and its doped forms and (nano)composites, were used in most previous studies. Despite their efficiency, the synthesized composites are relatively expensive and may be toxic for biota; therefore, their full-scale application represents a challenging task. In this regard, the naturally occurring minerals, which are active in the UV/VIS spectrum range and suppliable in large quantities, have gained increasing attention as cost-effective photocatalysts for water disinfection [4,5]. To date, natural sphalerite [6,7,8,9] and pyrite [10] were proven to be effective for photocatalytic inactivation of pathogenic microorganisms in water. However, there is still a limited number of studies on photocatalytic degradation of organic pollutants using natural photoactive minerals. Specifically, the natural sphalerite from Nigeria [11] and China [12] showed a photocatalytic activity towards degradation of methyl orange dye. Recently, Li et al. (2020) [13] also reported a photocatalytic activity of natural wolframite under visible light for degrading methylene blue and inactivating *E. coli*. To the best of our knowledge, natural sonophotoactive minerals have not been used in SPC for degradation of organic pollutants so far. Furthermore, low-frequency ultrasound (<100 kHz, typically 20–45 kHz) was commonly employed in SPC, whereas the potential of high-frequency ultrasound (>100 kHz and MHz range) remains much less investigated. Meanwhile, high-frequency ultrasound appears to be more beneficial than the low-frequency ultrasound due to increased ROS generation [14,15]. Therefore, the present study aimed at characterizing and evaluating the sonophotocatalytic activity of natural sphalerite (NatS) under high-frequency ultrasonic (1.7 MHz) and ultraviolet light-emitting diodes (365 nm) irradiation. To enhance the sonophotocatalytic activity, it was calcined at different temperatures. 4-chlorophenol was used as a model organic pollutant in degradation experiments.

## 2. Materials and Methods

### 2.1. Chemicals

4-chlorophenol (4-CP, 99%, Sigma-Aldrich, St. Louis, MO, USA), *p*-chlorobenzoic acid (*p*CBA, 99%, Acros, Geel, Belgium), hydrogen peroxide (33%, Lega, Dzerzhinsk, Russia), sodium bicarbonate (Khimreaktivsnab, Ufa, Russia), 4-aminoantipyrine and potassium ferricyanide (Vekton, St Petersburg, Russia) were used as supplied. Stock solutions of 4-CP and H_2_O_2_ were prepared in deionized water (18.2 mΩ·cm) produced by a Simplicity^®^UV system from Millipore (Burlington, MA, USA).

### 2.2. Catalyst Preparation

The raw natural sphalerite used in this study was collected from Dovatka deposit in Buryatia Republic, Russia (Geological Institute SB RAS, Ulan-Ude, Russia). The sample was crushed on ball mill, manually ground in the agate mortar and, finally, sieved to obtain powder with the particle sizes below 75 μm. The natural sphalerite was also calcined in muffle furnace (SNOL-1150, AB Umega, Ukmergė, Lithuania) at 500, 900 and 1200 °C for 5 h each. The calcined catalyst was ground again before each degradation experiment.

### 2.3. Treatment Procedure

**Figure 1:**
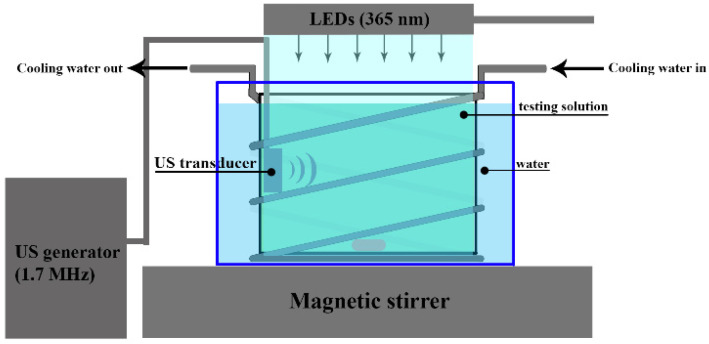
A scheme of sonophotoreactor.

Experiments were performed in an open glass batch sonophotoreactor under magnetic stirring (IKA, Staufen, Germany) (Figure 1).

A UVA LED array (Yonton, model YT-100WUV370-0, 100 W, Shenzhen Yonton Opto Co., Shenzhen, China), emitting at 365 nm, was positioned above the reactor at a distance of 1.5 cm from the solution surface. The incident intensity was 28 mW/cm^2^ across the UVA spectrum, as estimated from the ferrioxalate actinometry data using an adapted protocol described previously [16]. A 1.7 MHz ultrasound frequency was applied through an orthogonally positioned transducer, which operated with an ultrasonic amplifier (Thu UL 42 E, 20 W, Hangzhou Lanben Trade Co., Hangzhou, China). The estimated ultrasound intensity was 0.2 W/cm^3^. The 4-CP aqueous solution at a natural pH of 5.8 (100 mL, C_0_ = 10 mg/L) was placed in a reactor, the catalyst powder (1 g/L) was added and the solution was stirred without any exposure (dark conditions) for 30 min to reach the adsorption equilibrium. Afterwards, it was irradiated with UVA LED and/or ultrasound in the presence and absence of H_2_O_2_. In the case of the H_2_O_2_-assisted system, it was added freshly prior to irradiation to obtain the final concentration in the range of 1–10 mM. Samples were withdrawn before and after exposure, centrifuged at 4000 rpm for 5 min (if a catalyst added) and analyzed for residual concentration. The temperature of solution was kept at 20 ± 2 °C by circulating cooling water through copper tubes. Due to the relatively high power, the UVA LED array was also thermostated by a water jacket. •OH probing experiments in the photocatalytic systems were conducted using 20 μM *p-*chlorobenzoic acid (*p*CBA) as a reference compound. Degradation experiments were conducted under the following conditions (Table 1).

### 2.4. Analysis

The elemental composition of raw sphalerite was determined using a scanning electronic LEO-1430VP microscope (Carl Zeiss, Oberkochen, Germany) with an energy-dispersive INCA Energy 350 spectrometer (Oxford Instruments, Abingdon, UK). The phase composition of raw and calcined sphalerite was determined by powder X-ray diffraction (PXRD) analysis using D2 Phaser (Cu*K*_α_ radiation, 1.5418 Å, Bragg–Brentano geometry, scintillation detector, Bruker, Karlsruhe, Germany). PXRD data were collected over the 5°–80° 2Θ range with step 0.02°. The PXRD patterns were checked using JCPDS PDF-2 Database. The samples of raw and calcined sphalerite were imaged by SEM (JCM-6000, Jeol, Tokyo, Japan).

The BET specific surface area of samples was measured using the method of low-temperature adsorption–desorption of nitrogen (ThermoSorb LP, Institute of Semiconductor Physics SB RAS, Novosibirsk, Russia). *p*CBA in radical probing experiments was determined by HPLC (Agilent 1260 Infinity chromatograph, Zorbax SB-C18 column) with a diode-array detector. It was eluted with a mixture of CH_3_OH and 1% CH_3_COOH (70:30) and detected at 230 nm. 4-CP in degradation experiments was determined by standard colorimetric method with 4-aminoantipyrine and potassium ferricyanide [17] using a Shimadzu UV-1800 spectrophotometer (Kyoto, Japan). The degradation efficiency (%) was calculated by Equation (1) [18]:

(1) $$\mathrm{Degradation}\;\mathrm{efficiency}=\frac{\left(C_{0}-C\right)}{C_{0}}\times 100$$

where *C*_0_ and *C* are concentrations before and after treatment, respectively.

## 3. Results and Discussion

### 3.1. Characteristics of Raw and Calcined Natural Sphalerite

The elemental analysis of raw natural sphalerite confirmed that the main elements were zinc and sulfur (95.7%) (Table 2). The sample also contained iron and trace amounts of manganese and cadmium.

**Figure 2:**
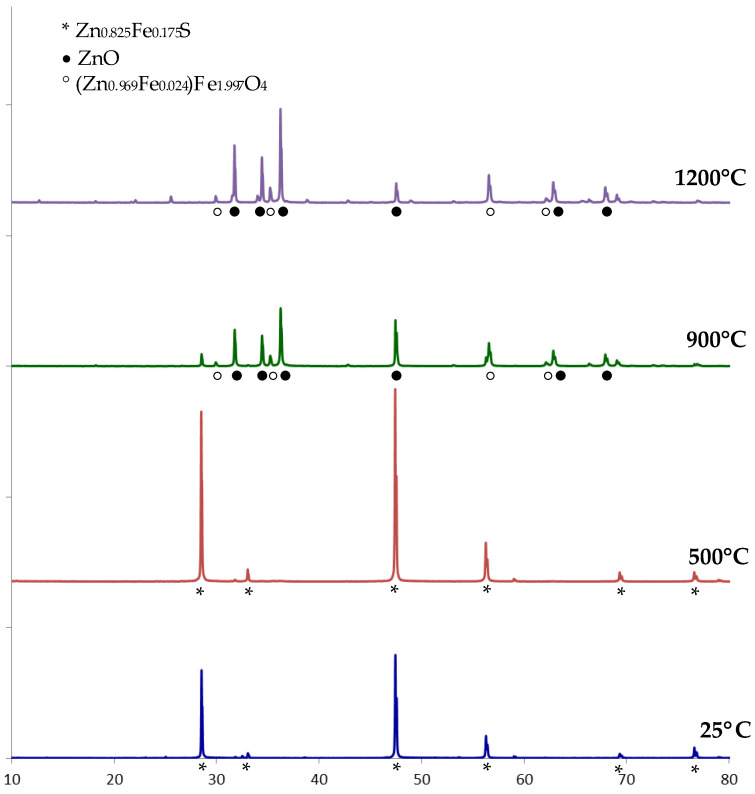
XRD patterns of raw (25 °C) and calcined natural sphalerite (500, 900 and 1200 °C).

The XRD pattern of raw natural sphalerite displayed prominent peaks at 2Θ angles of 28.5°, 47.3°, 56.2°, which can be well indexed to the mineral phase of Zn_0_._825_Fe_0_._175_S (JCPDS card no. 01-089-4936, Sphalerite, ferroan) (Figure 2). After calcination at 500 °C, the characteristic peaks of sphalerite remained in the XRD pattern. The peaks of two phases, ZnO (JCPDS card no. 01-089-7102) and (Zn_0_._969_Fe_0_._024_)Fe_1_._997_O_4_ or 0.969ZnO·0.024FeO·0.9985Fe_2_O_3_ (JCPDS card no. 01-070-3377, Franklinite), were observed in the XRD pattern of the sample calcined at 900 °C. The same phases of ZnO and (Zn_0_._969_Fe_0_._024_)Fe_1_._997_O_4_ were identified after calcination at 1200 °C (Figure 2).

Thus, raw natural sphalerite at 900 and 1200 °C was converted to zinc oxide and franklinite. It is known that zinc oxide and sulfur dioxide (IV) are formed under high-temperature calcination of zinc sulfide in air atmosphere (2):

(2) $$2\mathrm{ZnS}+3O_{2}\;\overset{t{}^{\circ}C}{\to }\;2\mathrm{ZnO}+2{\mathrm{SO}}_{2}$$

**Figure 3:**
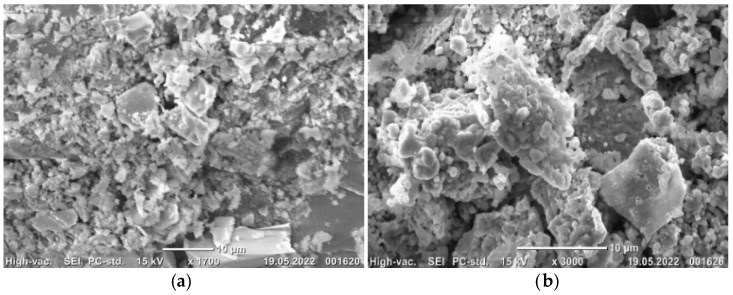
SEM images of raw (**a**) and calcined at 900 °C (**b**) natural sphalerite.

ZnO was also previously identified in the calcined natural sphalerite [5,11] and synthesized zinc sulfide [19]. The measured BET specific surface area of raw and calcined at 900 °C natural sphalerite was 0.46 and 3.0 m^2^/g, respectively. Such values are close to powdered natural wolframite (0.9763 m^2^/g) [13]. The surface of raw sample is represented by irregularly shaped micro-particles in the wide range of sizes, whereas the calcined sample displayed mainly the agglomerates of grains with a particle size less than 10 μm (Figure 3). There were also some voids between these agglomerates that may provide a higher surface area.

### 3.2. Sonophotocatalytic Degradation of 4-Chlorophenol

**Figure 4:**
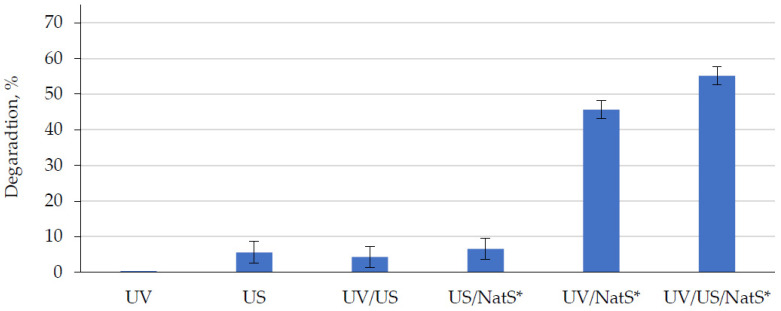
4-chlorophenol degradation under UVA LED (365 nm) and/or ultrasonic (1.7 MHz) irradiation using calcined natural sphalerite (NatS*, 900 °C, 1 g/L, 75 μm sieve) [4-CP]_0_ = 10 mg/L, exposure time = 1 h.

No degradation was observed after stirring in the presence of catalyst without any exposure for 30 and 120 min. The sorption effect was negligible (~1%). 4-CP was also resistant to direct UV photolysis and ~6% was degraded after ultrasonication only. Simultaneous exposure to UV and US radiation did not enhance the degradation efficiency (Figure 4). Raw natural sphalerite showed no photocatalytic activity under the experimental conditions applied. The bandgap energy of natural sphalerite is 3.6 eV (345 nm) and decreases to 3.20 eV (388 nm) for natural ZnO (zincite) [5]. As such, sphalerite was examined towards its photocatalytic activity after calcination at 500, 900 and 1200 °C. The highest removal efficiency was found after 1 h UV exposure for the sample calcined at 900 °C in the raw: 900 °C (46%) > 1200 °C (14.5%) > 500 °C (4.6%). We assume that the formed ZnO, a major phase, is responsible for photocatalytic activity of calcined natural sphalerite (900 °C) with the excitation at 365 nm. When calcined at 1200 °C, the sample performed much lower efficiency, presumably, due to reduction of active sites number. In contrast, Li et al. (2018) [20] reported that the natural sphalerite from China, which was calcined at 1200 °C, was more photocatalytically active than the sample after calcination at 900 °C towards degradation of methyl orange dye, although the same phases of ZnO/ZnFe_2_O_4_ were present in both samples. Other natural sphalerite from Nigeria exhibited the best photocatalytic performance at 700 °C [11].

In our case, the natural sphalerite after calcination at 900 °C (NatS*) was selected for exploring its sonophotocatalytic activity. Additional ultrasonication increased the degradation efficiency by 10% (Figure 4). It is known that a synergy between sonocatalytic and photocatalytic processes in SPC is quantified through synergistic index [2,21], which can be calculated as follows (3):

(3) $$\mathrm{Synergistic}\;\mathrm{index}=\frac{{\%\;\mathrm{Degradation}}_{\mathrm{UV}/\mathrm{US}/\mathrm{Catalyst}}}{{\%\;\mathrm{Degradation}}_{\mathrm{UV}/\mathrm{Catalyst}}+{\%\;\mathrm{Degradation}}_{\mathrm{US}/\mathrm{Catalyst}}}$$

Result showed that a synergistic effect with index of 1.06 was statistically insignificant and an additive effect was observed at a given ultrasound intensity. Literature analysis revealed contradictory data. No synergy was also evident (as estimated) for the UV/US/ZnO system using pure nano-sized ZnO, low-frequency ultrasound (20 kHz) and low-pressure mercury lamp (254 nm) for decolorizing direct blue dye [22]; however, in their later study, the authors reported a synergistic effect with indices from 1.38 to 2.30 [23]. An additive effect was also reported for sonophotocatalytic degradation of diclofenac (Fe-ZnO + 213 kHz + Xe lamp with 320 and 420 nm cut-off filters) [24] and norflurazon (Au-ZnO + 42 kHz + ≥420 nm) [25]. Meanwhile, ZnO sonophotocatalysis synergistically accelerated the degradation of chrome intra orange G dye (30 kHz + 365 nm) [26]. Recent studies supported the existence of synergy in the ZnO-based sonophotocatalytic degradation of sulfamethoxazole (24 kHz + UVA LEDs) [27], rhodamine B (40/59 kHz + visible light) [28] and methyl orange dyes (40 kHz + Xe lamp) [29]. It should be emphasized that the above-cited studies dealt with synthesized ZnO-based composites.

**Figure 5:**
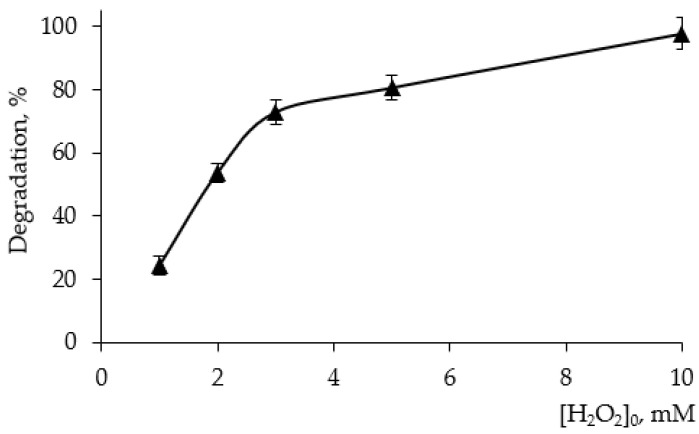
Effect of initial concentration of H_2_O_2_ on the efficiency of 4-chlorophenol degradation under UVA LED irradiation (365 nm). [4-CP]_0_ = 10 mg/L, exposure time = 1 h.

Considering the H_2_O_2_-assisted systems, initially, the effect of H_2_O_2_ concentration on the percentage degradation by UV/H_2_O_2_ process was studied (Figure 5).

Based on the obtained results, the lowest examined concentration of 1 mM (34 mg/L) was selected in order to obtain the measurable contribution of different processes in the hybrid sonophotocatalytic systems. This concentration also corresponds to stoichiometric molar ratio between 4-CP (0.078 mM) and H_2_O_2_ (1:13) in the hypothetical equation of mineralization (4):

C_6_H_4_(OH)Cl + 13H_2_O_2_ → 6CO_2_ + 15H_2_O + HCl (4)

**Figure 6:**
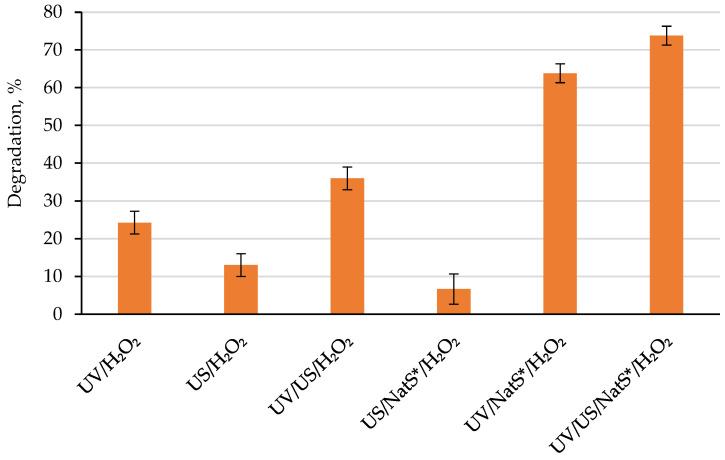
4-chlorophenol degradation under UVA LED (365 nm) and/or ultrasonic (1.7 MHz) irradiation using calcined natural sphalerite (NatS*, 900 °C, 1 g/L, 75 μm sieve) in the presence of 1 mM H_2_O_2_. [4-CP]_0_ = 10 mg/L, exposure time = 1 h.

Addition of 1 mM H_2_O_2_ improved the degradation to 64% in the system UV/NatS*/H_2_O_2_, providing an additive effect (Figure 6).

It is notable that the contribution of NatS* was ~2-fold higher than that of H_2_O_2_ (46 vs. 24%). However, simultaneous ultrasonic exposure did not significantly enhance the degradation in the system UV/US/NatS*/H_2_O_2_ (74%). This is presumed to be due to the prevalent contribution of photo-based processes (UV/H_2_O_2_ and UV/NatS*) as compared to sono-based processes. The hybrid UV/US/ZnO/H_2_O_2_ process was also previously applied for degrading Reactive Yellow azo dye [30] and antibiotic ofloxacin [31]. Note that the analytical grade pure ZnO, low-frequency ultrasound (20, 40 kHz) and conventional UVC low-pressure mercury lamp (254 nm) were applied in these studies. Patidar and Srivastava (2021) [31] reported a high synergistic effect in the UV/US/ZnO/H_2_O_2_ system with index of 3.10. Under our experimental conditions an additive effect was observed (index = 1.05).

**Figure 7:**
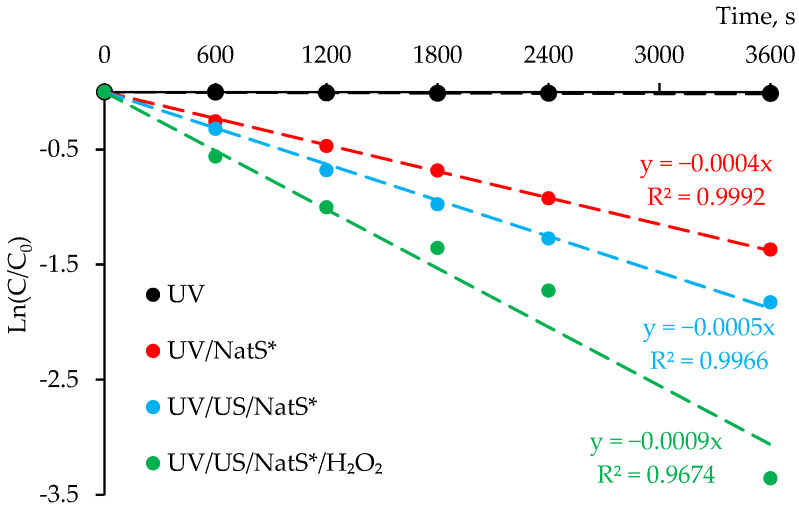
*p*CBA degradation plots under UVA LED (365 nm) and/or ultrasonic (1.7 MHz) irradiation using calcined natural sphalerite (NatS*, 900 °C, 1 g/L, 75 μm sieve). [*p*CBA]_0_ = 20 µM, [H_2_O_2_]_0_ = 1 mM.

Radical probing with *p*CBA also showed the sono- and photocatalytic activity of NatS* and indirectly confirmed •OH formation (Figure 7).

The steady-state concentration of hydroxyl radicals ([•OH]_ss_) was estimated from the slope of *p*CBA degradation plot (rate constant) according to Equation (5) [32]:

(5) $$-\frac{d\left[p\mathrm{CBA}\right]}{dt}=k_{p\mathrm{CBA},•\mathrm{OH}}\;[p\mathrm{CBA}][•\mathrm{OH}{]}_{\mathrm{ss}}$$

where *k_p_*_CBA,__•OH_ = 5 × 10^9^ M^−1^ s^−1^.

The efficiency of •OH formation in the selected systems increased in the order: UV < UV/NatS* < UV/US/NatS* < UV/US/NatS*/H_2_O_2_ and the estimated concentrations [•OH]_ss_ were 0.8 × 10^−13^, 1.0 × 10^−13^ and 1.8 × 10^−13^ M, respectively. At a large scale, a high intensity ultrasound can be applied for enhancing radical generation and attaining the synergistic effect. Besides, ultrasonication is beneficial for treating turbid waters due to much lower absorption as compared to UV radiation.

## 4. Conclusions

In this study, high-frequency ultrasound (1.7 MHz) was applied to enhance the photocatalytic degradation of 4-chlorophenol under UVA LED (365 nm) irradiation using natural sphalerite after calcination at 900 °C. Results revealed a sonophotocatalytic activity of calcined natural sphalerite, reaching 55% degradation of 4-chlorophenol after 1 h exposure as compared to 45% without ultrasonication. However, no synergy between sono- and photocatalytic processes was found. Addition of H_2_O_2_ significantly increased the efficiency of photocatalytic and sonophotocatalytic degradation by ~27%; however, an additive effect was also observed.

In contrast to synthesized ZnO-based photocatalysts, the natural sphalerite from Dovatka deposit (Siberia) exhibited the highest efficiency after calcination at 900 °C.

Application of high intensity ultrasound at large scale can be recommended for making the contribution of sonocatalysis more pronounced. In conclusion, heterogeneous sonophotocatalysis, which combines high-frequency ultrasound (1.7 MHz) and UVA/VIS light in the presence of natural sonophotocatalyst and H_2_O_2_, can be considered as a promising strategy for further investigating the degradation of organic pollutants in water.

## Figures and Tables

**Table 1:** Oxidation systems used in the study.

No H_2_O_2_	With H_2_O_2_
UV	UV + H_2_O_2_
US	US + H_2_O_2_
UV + US	UV + US + H_2_O_2_
UV + NatS ^1^	UV + NatS + H_2_O_2_
US + NatS	US + NatS + H_2_O_2_
UV + US + NatS	UV + US + NatS + H_2_O_2_

^1^ natural sphalerite.

**Table 2:** Elemental composition of raw natural sphalerite.

Element	Fe	Mn	Zn	Cd	S
Wt. %	5.99	1.37	61.79	0.57	33.95

## Data Availability

Not applicable.
